# Data on projections of surface water withdrawal, consumption, and availability in the conterminous United States through the 21st century

**DOI:** 10.1016/j.dib.2019.103786

**Published:** 2019-02-25

**Authors:** Kai Duan, Peter V. Caldwell, Ge Sun, Steven G. McNulty, Yang Zhang, Erik Shuster, Bingjun Liu, Paul V. Bolstad

**Affiliations:** a School of Civil Engineering, Sun Yat-Sen University, Guangzhou, China; b Department of Forest Resources, University of Minnesota, Saint Paul, MN, USA; c Coweeta Hydrologic Laboratory, USDA Forest Service, Otto, NC, USA; d Eastern Forest Environmental Threat Assessment Center, USDA Forest Service, Raleigh, NC, USA; e Department of Marine, Earth, and Atmospheric Sciences, North Carolina State University, Raleigh, NC, USA; f National Energy Technology Laboratory, US Department of Energy, Pittsburgh, PA, USA

**Keywords:** Surface water, Water withdrawal, Water consumption, Water supply, Hydrologic system, United States

## Abstract

We report data on the projections of annual surface water demand and supply in the conterminous United States at a high spatial resolution from 2010s to the end of the 21st century, including: 1) water withdrawal and consumption in the water-use sectors of domestic, thermoelectric power generation, and irrigation; 2) availability of surface water generated from local watershed runoff, accumulated from upstream areas, and artificially transferred from other basins. These data were derived from the projected changes in climate, population, energy structure, technology and water uses. These data are related to the original article “Understanding the role of regional water connectivity in mitigating climate change impacts on surface water supply stress in the United States” (Duan et al., 2019) [1].

**Table:** Specifications table

Subject area	*Water resources*
More specific subject area	*Hydrology, Water management*
Type of data	*Table*
How data was acquired	*Survey, modeling*
Data format	*Analyzed*
Experimental factors	*Observations and projections of related natural and environmental factors were compiled and processed to produce data on the aspects of water demand and water supply*
Experimental features	*Continuous time series data of water withdrawal and consumption in domestic use, thermoelectric power generation, and crop irrigation, and available surface water supply across over two thousand watersheds under the influence of climatic and socioeconomic changes*
Data source location	*Conterminous United States*
Data accessibility	*Data are with this article*
Related research article	*K. Duan, P.V. Caldwell, G. Sun, S.G. McNulty, Y. Zhang, E. Shuster, B. Liu, P.V. Bolstad, Understanding the role of regional water connectivity in mitigating climate change impacts on surface water supply stress in the United States, J Hydrol, 570 (2019) 80–95.*

**Table:** 

**Value of the Data** • The data provide a national projection of natural water balance and anthropogenic water uses and the linkage between them in a changing world • The data provide a reference for water managers and stakeholders at various spatial levels • The data can assist further research on a broad range of disciplines, e.g., hydrology, geosciences, agriculture, economy, water management.

## 1 Data

This paper reports data of annual surface water withdrawal, consumption, and availability across 2099 8-digit Hydrologic Unit Code watersheds in the conterminous United States from 2010s to the end of the 21st century. These data were produced based on survey datasets in historical periods and projected changes in population, energy use, climate, and technology through the 21st century that are associated with the U.S. Department of Energy's (DOE) electric power plans and the Intergovernmental Panel on Climate Change's (IPCC) scenarios of climatic, demographic, and socio-economic developments. Data on water demand for off-stream use and the corresponding water consumption include three major water-use sectors, i.e., thermoelectric, irrigation, and domestic, and the sum of all sectors. Data on the aspect of water availability include the water generated from local watershed runoff, the water accumulated in river channels from upstream areas, and the water artificially transferred from other basins.

We reconciled the datasets of population, electricity generation, climate, and water uses from different sources to provide a projection of total water demand and total water availability. Two scenarios were compiled to facilitate assessment on regional water scarcity and vulnerability, i.e., Intermediate Stress (IS) scenario and High Stress (HS) scenario. The IS scenario was driven by population under IPCC's B1 scenario, climate under the Representative Concentration Pathways (RCP) 4.5 scenario, and power generation with the Clean Power Plan proposed by the U.S. Environmental Protection Agency (EPA), while the HS scenario was driven by population under IPCC's A2 scenario, climate under the RCP 8.5 scenario, and power generation without the Clean Power Plan. These two scenarios of water stress level are closely related to climate change and the associated mitigation measures, and thus can be used to represent a future with and without climate change mitigation strategies, respectively. Readers are referred to Duan et al. [1] for more details.

## 2 Experimental design, materials, and methods

### 2.1 Water withdrawal and water consumption

In 2010, the three major water-use sectors accounted for 84% of the total surface freshwater withdrawal, i.e., 50% thermoelectric, 28% irrigation, and 6% domestic [2]. Thus, we have focused on the potential changes in these sectors in the future and assumed water uses in other sectors to remain constant. Projection of future water withdrawal and consumption was based on the extrapolation of past trends and the estimates of demographic, economic, and climatic forces on water uses.

Domestic water withdrawal/consumption was estimated as population multiplying the per capita withdrawal/consumption in 2010. Future changes in population was obtained from the Integrated Climate and Land-Use Scenarios v1.3 dataset provided by the U.S. EPA.

Thermoelectric water withdrawal was estimated as total thermoelectric power generation multiplying per kWh water use, and water consumption as water withdrawal multiplying water consumption per unit withdrawal. The electric power projections in 2010–2040 were obtained from the Annual Energy Outlook provided by the Energy Information Administration (EIA) of DOE, and then linearly extrapolated forward from 2041 to 2099 using the average rates of change in 2010–2040 for each Electricity Market Module region. The rates of per kWh water use and consumption per unit withdrawal were estimated based on the projections of additions and retirements of thermoelectric generating capacity and cooling systems conducted by the National Energy Technology Laboratory (NETL) [3].

Irrigation water demand was calculated as irrigated area multiplying irrigation use per unit area (i.e., irrigation efficiency). Future changes in irrigated area and irrigation efficiency due to socioeconomic causes (e.g., policy, market, technology) were obtained from Brown et al. [4], and the additional impacts of climate change on irrigation efficiency were simulated using the model suggested by Döll [5]. Irrigation water consumption were projected based on the assumption that water consumption per unit area would remain at the same levels as in 2010 in irrigated areas, i.e., water consumption per unit withdrawal would increase accordingly as the updates of irrigation system decrease withdrawal per unit area.

### 2.2 Water availability

The total available surface water supply for a watershed (total water flow, TF) was calculated as the sum of local water flow (LF), upstream water flow (UF) minus upstream water consumption (UWC), and inter-basin water transfer (IBT): TF = LF + UF − UWC ± IBT. LF was simulated monthly at watershed level by driving the Water Supply Stress Index model with climate data under the scenarios of RCP 4.5 and RCP8.5 [6]. UF was calculated by summarizing the LFs in connected upstream watersheds, and UWC was the total amounts of water consumption in all the water-use sectors in these areas. The multi-model mean results of LF, UF, and UWC derived from climate outputs of 19 Global Climate Models (GCMs) are available with this article. Records of a total of 228 IBTs across the country were obtained from Emanuel et al. [7], and then the impacts of these transfers on regional water availability were simulated based on upstream-downstream connections across the country.
