# Supplementary material for: Data on projections of surface water withdrawal, consumption, and availability in the conterminous United States through the 21st century
Source: Data Brief. 2019 Feb 25;23:103786. doi: 10.1016/j.dib.2019.103786 (PMC6661235; doi:10.1016/j.dib.2019.103786)
Supplement: Multimedia component 2 [file mmc2.doc]

<ce:appendices><ce:section id="appsec1" view="extended"><ce:section-title id="sectitle0045">Transparency document</ce:section-title><ce:para id="p0070">The following is the transparency document related to this article:<ce:display><ce:e-component id="mmc1"><ce:label>Multimedia component</ce:label><ce:alt-text role="short" id="alttext0010">Multimedia component</ce:alt-text><ce:link locator="mmc1" xlink:type="simple" xlink:role="http://data.elsevier.com/vocabulary/ElsevierContentTypes/46.1" xlink:href="pii:S2352340919301374/mmc1"/></ce:e-component></ce:display></ce:para></ce:section><ce:section id="appsec2" view="compact-standard"><ce:section-title id="sectitle0050">Transparency document</ce:section-title><ce:para id="p0075">Transparency document associated with this article can be found in the online version at <ce:inter-ref xlink:href="https://doi.org/10.1016/j.dib.2019.103786" id="intref0010">https://doi.org/10.1016/j.dib.2019.103786</ce:inter-ref></ce:para></ce:section><ce:section id="appsec3" view="extended"><ce:label>Appendix A</ce:label><ce:section-title id="sectitle0055">Supplementary data</ce:section-title><ce:para id="p0080">The following is the Supplementary data to this article:<ce:display><ce:e-component id="mmc2"><ce:label>Multimedia component 2</ce:label><ce:alt-text role="short" id="alttext0015">Multimedia component 2</ce:alt-text><ce:link locator="mmc2" xlink:type="simple" xlink:role="http://data.elsevier.com/vocabulary/ElsevierContentTypes/46.1" xlink:href="pii:S2352340919301374/mmc2"/></ce:e-component></ce:display></ce:para></ce:section><ce:section id="appsec4" view="compact-standard"><ce:label>Appendix A</ce:label><ce:section-title id="sectitle0060">Supplementary data</ce:section-title><ce:para id="p0085">Supplementary data to this article can be found online at <ce:inter-ref xlink:href="https://doi.org/10.1016/j.dib.2019.103786" id="intref0015">https://doi.org/10.1016/j.dib.2019.103786</ce:inter-ref>.</ce:para></ce:section></ce:appendices>
